# Inferring slowly-changing dynamic gene-regulatory networks

**DOI:** 10.1186/1471-2105-16-S6-S5

**Published:** 2015-04-17

**Authors:** Ernst C Wit, Antonino Abbruzzo

**Affiliations:** 1 Johann Bernoulli Institute, University of Groningen, Nijenborgh 9, 9747 AG Groningen, Netherlands; 2 SEAS - Dipartimento di Scienze Economiche Finanziarie e Statistiche, Università degli Studi di Palermo, Viale delle Scienze Ed., 13, 90128 Palermo, Italy

**Keywords:** gene-regulatory networks, graphical models, *L*_1 _penalized inference

## Abstract

Dynamic gene-regulatory networks are complex since the interaction patterns between their components mean that it is impossible to study parts of the network in separation. This holistic character of gene-regulatory networks poses a real challenge to any type of modelling. Graphical models are a class of models that connect the network with a conditional independence relationships between random variables. By interpreting these random variables as gene activities and the conditional independence relationships as functional non-relatedness, graphical models have been used to describe gene-regulatory networks. Whereas the literature has been focused on static networks, most time-course experiments are designed in order to tease out temporal changes in the underlying network. It is typically reasonable to assume that changes in genomic networks are few, because biological systems tend to be stable.

We introduce a new model for estimating slow changes in dynamic gene-regulatory networks, which is suitable for high-dimensional data, e.g. time-course microarray data. Our aim is to estimate a dynamically changing genomic network based on temporal activity measurements of the genes in the network. Our method is based on the penalized likelihood with $\ell_{1}$-norm, that penalizes conditional dependencies between genes as well as differences between conditional independence elements across time points. We also present a heuristic search strategy to find optimal tuning parameters. We re-write the penalized maximum likelihood problem into a standard convex optimization problem subject to linear equality constraints. We show that our method performs well in simulation studies. Finally, we apply the proposed model to a time-course T-cell dataset.

## Introduction

A single microarray experiment provides a snapshot of the expression of many genes simultaneously under a particular condition. Gene expression is a temporal process, in which different genes are required and synthesized for different functions and under different conditions. Even under stable conditions, due to the continuous degradation of proteins, mRNA is transcribed continuously and new proteins are generated. This process is highly regulated. In many cases, the expression programme is initiated by the activation of a few transcription factors, which in turn activate many other genes that react in response to the newly arisen conditions. Transcription factors are proteins that bind to specific DNA sequences, thereby controlling the flow of genetic information from DNA to mRNA. For example, when cells are faced with a new external environment, such as starvation [1], infection [2] or stress [3], they react by activating a particular expression program. Taking a snapshot of the expression profile following a new condition can reveal some of the genes that are specifically expressed under the new condition. However, in order to discover the interaction between these genes, it is necessary to measure the genes across time in a time-course expression experiment. These temporal measurements potentially allow us to determine not only the stable state following a new condition, but also the gene interactions that were activated in order to arrive at this new state. The biological and computational issues involved in designing and analyzing gene expression data in general, and time-course expression data in particular, is discussed in [4].

In this paper, we propose a graphical model for describing temporal interaction patterns between genes. Graphical models explore conditional independence relationships between random variables. They can be divide into directed graphical models, e.g. Bayesian networks [5,6], undirected graphical models, e.g. Gaussian graphical models [7,8] and mixed versions, such as chain graphical models [7]. Bayesian networks have been successfully used to describe certain types of gene-regulatory networks [9]. However, Bayesian networks suffer two major limitations. Firstly, they cannot be used to describe cyclic graphs. This rules out using them for describing simultaneous feedback loops in gene regulatory networks. Secondly, they perform poorly on sparse microarray data as shown by [10]. It is possible to "unroll" cycles into spirals through time, so the first limitation can partially be overcome [11-13]. Instead, we propose to model such cycles more directly as undirected edges in our conditional independence graph. Furthermore, our method will allow for "directed" edges between consecutive time points.

The class of Gaussian graphical models (GGM) have been particularly popular. The main advantage for GGMs is that the precision matrix, i.e. the inverse of the covariance matrix, can be used to "read off" the conditional independence relationships between the random variables. The literature on estimating the inverse covariance matrix goes back to [14], who also introduced hypothesis testing approaches to determining whether particular elements of the inverse covariance matrix are zero. The more zeroes in the inverse covariance matrix, the sparser the underlying conditional independence graph.

Regulatory elements in genetic networks are highly structured. In order to guarantee an appropriate response to a particular change in the environment, most gene interactions are highly specific. The detailed molecular structure of genes and gene products are responsible for this level of specificity. Another biological requirement is that gene regulation is fast in reacting to changes in the environment. Heat shocks should almost instantaneously result in an adaptive response from the yeast cell. From this point of view signals should be able to travel fast through the gene regulatory network: the network should have a small world property. Consequently, most gene regulatory networks are sparse small-world graphs. If the expression of the genes can be assumed to be normally distributed, then this means that most of the elements in the precision matrix are equal to zero. A standard approach in statistical modelling to identify zeroes in the precision matrix is the backward stepwise selection method, which starts by removing the least significant edges from a fully connected graph, and continues removing edges until all remaining edges are significant according to individual partial correlation tests. A conservative simultaneous testing procedure was proposed by [15]. However, [16] showed that this two-step procedure, in which parameter estimation and model selection are done separately, can lead to instability: small perturbations in the data can result in completely different graph structures estimates.

[17] showed that $\ell_{1}$ penalized likelihood is a sensible way to introduce sparse solutions in a regression setting. The same idea can be used to estimate sparse Gaussian graphical models, i.e. to induce zeroes in the estimated inverse covariance matrix. By penalizing the likelihood by a multiple of the $\ell_{1}$-norm of the elements of the inverse of the covariance matrix results into exact zeroes in the penalized maximum likelihood estimate. The larger the value of this multiplicative tuning parameter the more zeroes will be estimated in the precision matrix. [18] introduced the $\ell_{1}$ penalized Gaussian graphical model and [19] showed that it is possible to select the tuning parameter in such a way as to control the familywise error rate. [20] introduced a fast and efficient algorithm to calculate the so-called graphical lasso solution. The graphical lasso estimates a single static network for a single condition. When there are multiple conditions, it may be sensible to presuppose a roughly common structure and jointly estimate common links across the graphs. [21] proposed a method that links the estimation of several graphical models through a hierarchical penalty. This graphical model leads to improvements compared to fitting separate models, since it borrows strength from other related graphs. Recently, [22] proposed a factorially coloured graph to estimate a common dynamic structure across time.

In this paper we propose a model to estimate slowly changing dynamic graphs using the $\ell_{1}$-regularization framework. The main idea is to impose the $\ell_{1}$-penalty not only on the inverse covariance matrix, but also on *changes *in the inverse covariance matrix over time. The new method is suitable for studying high-dimensional time-course gene activity data. In order to solve the penalized maximum likelihood problem, we take advantage of an efficient solver developed by [23] to solve the optimization problem with linear constraints. We propose a heuristic search algorithm to fix the tuning parameters, that regulate sparsity and dynamic changes in the networks.

The rest of this paper is organized as follows. The next section gives a description of our motivating example and a brief overview of Gaussian graphical models. In Methods, we describe the slowly changing dynamic network model and its estimation. In Results, we show the results of a simulation study and apply our method to the time-course T-cell dataset. Finally, we discuss the advantages of our method and point out further directions for development.

## Motivation: T-cell activation

T-cells are white bloods cell that play a central role in cell-mediated immunity. Activation of T-cells occurs through the simultaneous engagement of the T-cell receptor and a costimulatory molecule, like CD28 or ICOS. Both are required for the production of an immune response. The signalling pathways downstream from activation usually engages the PI3K pathway and the recruiting PH domain containing signaling molecules, like PDK1, that are essential for the activation of PKC-*θ*, and eventual IL-2 production. Although certain things are known about the structure of the T-cell pathway, its timing and its precise structure are still unknown.

Two cDNA microarray experiments were performed to collect gene expression levels for analyzing T-cell activation. Human T-cells coming from the cell line Jakart were cultured in a laboratory. When the culture reached a consistency of 10^6 ^cells/ml, the cells were treated with two treatments, the calcium ionosphere and the PKC activator phrorbolester PMA. This stimulation of the T-cells resulted in their activation. Gene expression levels for 88 genes were collected across 10 time points: the first one just before T-cell activation, at a nominal time-point 0, and 9 time points at 2, 4, 6, 8, 18, 24, 32, 48, 72 hours after T-cell activation. In the first experiment the microarray was divided such that 34 sub-arrays were obtained. Each of these 34 sub-arrays contained the strands of 88 genes under investigation. Strands are the complementary bases for the mRNA, which is the single-stranded transcribed copy of the DNA. In the second microarray experiment the microarray was dived into 10 sub-arrays. Each of these 10 sub-arrays contained the strands of the same 88 genes. Both microarray experiments used 10 different slides to collect the 10 temporal measurements. The experiment is described in detail in [24].

Two further steps were conducted by [24] to obtain a set of genes that were highly expressed and normalized across the two microarray experiments. Firstly, genes with high variability between the two microarrays and within the same time point were removed. No further information is available about the minimum level of reproducibility they adopted. According to [24], thirty-one genes were to be removed since they did not show enough reproducibility. Secondly, normalization methods were applied to remove systematic variation due to experimental artifacts. The normalization method used is described in [25].

At this point we assume that the 44 sub-array replicates are independent samples and that the temporal replicates across these sub-arrays are functionally dependent replicates. These two assumptions result in a dataset of 44 independent replicates across 57 genes and 10 time points.

## Methods

In this section, we describe the model that we adopt in order to study the underlying time-varying genomic network for the T-cell data. We argue that time-course datasets should be analyzed in a way, that is sensitive to the underlying biology. If one does not use a model that is able to describe a time-varying network, there would not have been a point in performing a time-course experiment. The bioinformatic tools should be adjusted to the needs of the biologist, who wants to infer particular aspects of the system. In this section, we first introduce a general graphical model. Secondly, we extend this model to the slowly changing graphical lasso model. Finally, we describe the computational details of performing penalized maximum likelihood.

### Gaussian graphical model

A graphical model is a tuple (*G*, P), where *G *= (*V, E*) is a graph with edges *E *that describe the conditional independence relationships of probability measure P on the vertices *V*. This means that one can use the graph *G *to read off the functional relationships between the random variables associated with the vertices. In particular, for any triple (*A, B, S*) of disjoint subsets of *V *such that *S *separates *A *from *B *in *G*, we have that for *Y *~ ℙ,

$${\text{Y}}_{A}{⊥}_{\mathbb{P}}\;{\text{Y}}_{B}|{\text{Y}}_{S}|.$$

This so-called global Markov property in turn implies the local and pairwise Markov properties.

In this paper, we will assume that the gene activity data *Y_i _*has a multivariate normal distribution, i.e., *Y *~ ℙ_***μ***,**Σ**_, with mean ***μ ***and covariance matrix **Σ**. Together with conditional independence graph *G *= (*V, E*), (*G*, ℙ_*μ*,Σ_) constitutes a Gaussian graphical model or a covariance selection model [14]. This Gaussian graphical model puts some conditions on the covariance matrix Σ. Let Θ = Σ^-1 ^be the precision or concentration matrix, then Θ contains all conditional independence information for the Gaussian graphical model. In particular,

$$\theta_{ij}=0\Leftrightarrow \left(i,j\right)\notin E\Leftrightarrow Y_{i}⊥Y_{j}|Y_{-\left\{i,j\right\}}.$$

In fact, it is easy to show that given the set of *E^c ^*= {(*i, j*) |*θ_ij _*= 0}, a multivariate normal probability distribution *f*(**y**) can be factorized as a product of functions *f *which do not jointly depend to *y_i _*and *y_j _*when (*i, j*) ∈ *E^c^*.

Given a set of *n *observations on the Gaussian graphical model, *y*_1_,...,*y_n_*, the log-likelihood can be written as

$$l\left(\mu ,\text{Θ}\right)=\frac{n}{2}\left\{\text{log}|\text{Θ}|-\text{Tr}\left(S\text{Θ}\right)-{\left(\mu -\bar{y}\right)}^{t}\text{Θ}\left(\mu -\bar{y}\right)\right\},$$

where $S=\sum_{k}\left(y_{k}-\bar{y}\right){\left(y_{k}-\bar{y}\right)}^{t}\text{/}n$ is the sample covariance matrix. From the form of the likelihood, it is clear that $\hat{\mu }=\bar{y}$ is the maximum likelihood estimate of *μ *irrespective of the number of observations and the underlying graph *G*. For the MLE for Θ, the story is more complicated. For the complete graph, the maximum likelihood estimate is not uniquely defined when the number of observations is less than the number of vertices, *n *< |*V*|. This situation is really common for experiments to infer genomic networks. On the other hand, a gene-regulatory network is typically sparse, which means that the number of links is small with respect to the possible number of connections. This may mean that Θ is estimable with respect to the underlying true sparse graph, *G*. The only problem is that we don't know which sparse graph that is. Therefore, we impose an additional constraint,

$${\hat{\text{Θ}}}_{\rho }=\text{arg}\underset{\text{Θ}}{\text{max}}\;l\left(\bar{y},\text{Θ}\right),$$

subject to

$$||\text{Θ}|{|}_{1}:=\overset{p-1}{\underset{i=1}{\sum }}\overset{p}{\underset{j=i+1}{\sum }}|\theta_{ij}|<\rho ,$$

where typically we do not penalize the diagonal of the precision matrix. Sparsity of the genomic network is not only our current best knowledge of the gene-regulatory system, but coincidently it is also computationally useful. [26] formally defines a graph *G *= (*V, E*) to be sparse, if |*E*| = *O*(|*V*|), where |*V*| is the number of vertices and |*E*| is the number of edges. A graph *G *is said to be dense, if |*E*| = *O*(|*V*|^2^). By constraining the estimate to satisfy an $\ell_{1}$ constraint, it is possible to combine estimation of the precision matrix Θ with the estimation of the underlying graph *G*.

### Dynamic Gaussian graphical models

In this section, we introduce the concept of a dynamic Gaussian graphical model, which extends the static Gaussian graphical model that was introduced in the previous section. We first define a dynamic graph *G *= (*V, E*). Consider a set of genes Γ = {*γ*_1_,..., *γ*_*p*_} and a set of time points where these genes were observed *τ *= {*t*_1_,..., *t_T_*}. We define the vertices of the dynamic graph as the Cartesian product of the genes and time points, *V *= Γ × *τ*. Therefore, a vertex in this graph is an element (*γ_g_, t_s_*). The edges are some subset of the Cartesian product of vertices, *E *⊂ *V *× *V*. An element of *E *will be written as {(*γ_g_, t_s_*), $\left(\gamma_{g\prime },t_{s\prime }\right)$}, stressing the fact that it links one gene at a particular time point with another gene at another time point. We will associate with each node of the graph a random variable *Y_gs_*, which represents the amount of gene activity of gene *g *at time *s*.

With the above ingredients, we can now define a **dynamic Gaussian graphical model **as the tuple (*G*, ℙ_*μ*,Θ_), where *G *= (Γ × *τ, E*) is a dynamic graph and ℙ_*μ*, Σ _is a collection of multivariate Gaussian distributions with mean *μ *and inverse covariance matrix Θ, that are compatible with the conditional independence relationships described in the edge set *E*.

In principle, the ordering of the vertices is arbitrary. For interpretation purposes, it helps to sort the vertices by time points and within time points by genes. This results in a natural partition {(*N_l_, S_l_*) |*l *= 0,..., *t *- 1} of the inverse covariance matrix Θ,

$$\text{Θ}=\left[\begin{array}{ccccccc} \begin{array}{cc} S_{0}^{1} & N_{0}^{1}\\ & S_{0}^{1} \end{array} & & \begin{array}{cc} S_{1}^{1} & N_{1}^{1}\\ N_{1}^{1} & S_{1}^{1} \end{array} & & \begin{array}{cc} S_{2}^{1} & N_{2}^{1}\\ N_{2}^{1} & S_{2}^{1} \end{array} & & \begin{array}{cc} ... & ...\\ ... & ... \end{array}\\ & & \begin{array}{cc} S_{0}^{2} & N_{0}^{2}\\ & S_{0}^{2} \end{array} & & \begin{array}{cc} S_{1}^{2} & N_{1}^{2}\\ N_{1}^{2} & S_{1}^{2} \end{array} & & \begin{array}{cc} S_{2}^{2} & N_{2}^{2}\\ N_{2}^{2} & S_{2}^{2} \end{array}\\ & & & & \begin{array}{cc} S_{0}^{3} & N_{0}^{3}\\ & S_{0}^{3} \end{array} & & \begin{array}{cc} S_{1}^{3} & N_{1}^{3}\\ N_{1}^{3} & S_{1}^{3} \end{array}\\ & & & & & & \begin{array}{cc} \ddots & \vdots \\ & \ddots \end{array} \end{array}\right]$$

where *S_l _*represent the self-self interactions of the genes and *N_l _*the network interactions between the genes, at time lag *l*. The self-self interactions therefore represent the diagonal and subsequent off-diagonals of the matrix Θ, whereas *N_l _*are the diagonal blocks and subsequent off-diagonal blocks minus the diagonal. Each of these subsets can be further partitioned, as indicated by $S_{l}^{t}$ and $N_{l}^{t}$. In these sub-partitions, $S_{l}^{t}$ is the persistence of genes from time point *t *until time point *t *+ *l*. Similarly, $N_{l}^{t}$ are the network interactions between genes at time points *t *and *t *+ *l*.

As the full dynamic Gaussian graphical model is still heavily parameterized with a typically big *pT *× *pT *inverse covariance matrix, it makes sense to consider relevant model subclasses. It is for example not particularly likely that two genes are related across a large time lag, conditional on the intermediate states. We therefore define the **autoregressive Gaussian graphical model of order *k ***(*G*, ℙ_*μ*,Θ_, *AR*(*k*)) as a dynamic Gaussian graphical model (*G*, ℙ_*μ*,Θ_), for which

$$\forall l>k:N_{l}=S_{l}=0.$$

This model assumes that genes are conditionally uncorrelated for time lags larger than *k*. In practice, we typically consider *k *= 1 or *k *= 2, which from an interpretational point of view are most interesting. It is important to note that the autoregressive Gaussian graphical model is directly associated to a particular network structure *G*, which represents the conditional dependence graph of the random variables associated with the vertices of the graph.

### Slowly changing dynamic graphical models

The main question this paper wants to answer is how to infer a meaningful biological dynamic network from noisy data on the nodes, such as, e.g., RNA seq values or protein levels. The two features we will assume particularly relevant of a gene network are its *sparsity *and its *persistence*. DNA, RNA and proteins are very specific molecules that are capable of interacting, typically, with only a very limited number of other molecules. This means that a genetic network is highly structured and selective, and, therefore, characterized by a high degree of sparsity. As genetic interactions depend very much on the basic molecular structure of its constitutive parts, the potential to interact between various genes will typically not change over time, unless particular regime changes in the cell affect its thermo-dynamic properties. Interactions in the dynamic network *G *therefore tend to persist over time. We will show in this subsection how we can incorporate these two ideas, sparsity and persistence of the network, in the interferential objective function by means of a penalized likelihood function.

In the T-cell experiment, we assume we have 44 observations from the 57 × 10 dimensional autoregressive Gaussian graphical model of order *k *= 1. Not only do we want to infer a sparse network *G*, but also one for which the underlying network partitions $N_{l}=\left\{N_{l}^{1},.\;.\;.\;,\;N_{l}^{t},\;.\;.\;.\;,N_{l}^{T}\right\}\;\;\left(l=0,1\right)$ change only slowly across time. This requires an additional set of constraints in our maximum likelihood inference. In general, we assume we have *n *observations *y*_1_,..., *y_n_*, each coming from the autoregressive *k *dynamic Gaussian graphical model (*G*, ℙ_*μ*,Θ_, *AR*(*k*)).

Given two tuning parameters *ρ*_1 _and *ρ*_2_, we define a slowly changing dynamic network as the solution of the penalized maximum likelihood of the autoregressive *k *dynamic Gaussian graphical model,

(1) $$l\left(\mu ,\text{Θ}\right)=\frac{n}{2}\left\{\text{log}|\text{Θ}|-\text{Tr}\left(S\text{Θ}\right)-{\left(\mu -\bar{y}\right)}^{t}\Theta \left(\mu -\bar{y}\right)\right\},$$

subject to

(2) $$||\text{Θ}|{|}_{1}<\rho_{1}$$

(3) $$\overset{k}{\underset{l=0}{\sum }}\overset{T-1}{\underset{s=1}{\sum }}||N_{l}^{s}-N_{l}^{s+1}|{|}_{1}<\rho_{2}$$

(4) $$\forall l>k;N_{l}=S_{l}=0$$

Whereas the first constraint induces a generally sparse dynamic network, the second constraint penalizes large changes in the network coefficients, thereby inducing a slowly changing or persistent network through time. Therefore, the penalty parameters are directly related to the zero and persistence structure of the estimate ${\hat{\text{Θ}}}_{\rho 1,\rho 2}$ and, therefore, to the estimate of the dynamic genetic graph ${Ĝ}_{\rho 1,\rho 2}$.

Solving the above penalized maximization problem is an active field of research in optimization. We use the log determinant proximal point approximation method developed by [23]. Each constraint gets coded into a linear map. We consider *A*(Θ) = ||Θ||_1 _associated with constraint (2), $B\left(\text{Θ}\right)=\sum_{l=0}^{k}\sum_{s=1}^{T-1}||N_{l}^{s}-N_{l}^{s+1}|{|}_{1}$ associated with constraint (3) and *C_l_*(Θ) = (*S_l_*; *N_l_*) associated with constraint (4). This method introduces two sets of slack variables to deal with the two inequality constraints. The constraint optimization problem (1) is now written as:

$$\hat{\text{Θ}}:\underset{\text{Θ}}{\text{argmin}}\left\{-\text{log}|\text{Θ}|+\text{Tr}\left(\text{S}\text{Θ}\right)+\lambda_{1}{\text{v}}^{+}+\lambda_{1}{\text{v}}^{-}+\lambda_{2}{\text{w}}^{+}+\lambda_{2}{\text{w}}^{-}\right\}$$

$$\begin{array}{cc} \text{subject to}\; & \text{A}\left(\text{Θ}\right)-{\text{v}}^{+}+{\text{v}}^{-}=\text{0}\\ & \text{B}\left(\text{Θ}\right)-{\text{w}}^{+}+{\text{w}}^{-}=\text{0}\\ & {\text{C}}_{1}\left(\text{Θ}\right)=\text{0},\;\;l=0,.\;.\;.\;,k\\ & \text{Θ}≻0,{\text{v}}^{+},{\text{v}}^{-},{\text{w}}^{+},{\text{w}}^{-}\geq \text{0}, \end{array}$$

where *λ*_1 _and *λ*_2 _are functions of *ρ*_1 _and *ρ*_2 _respectively. In this format, the optimization can be solved directly by LogDetPPA.

The non-negative tuning parameters *λ*_1 _and *λ*_2 _effectively determine the sparsity and the persistence of the network through time, respectively. Selecting these tuning parameters is a form of model selection. Depending on the interests of the user, which can be maximizing posterior model probability or minimizing prediction error, either a BIC-type criterion or an AIC-type criterion is proposed. We consider a grid of values (*λ*_1_*, λ*_2_) and minimize information criterion scores such as AIC, AICc, and BIC. Then we use stability selection to select a more stable graph [27].

*Example: T-cell *We consider a subset of the T-cell data to illustrate the performance of the autoregressive Gaussian graphical model approach with a slowly changing network penalty. Only 4 genes and 2 time points were considered. Table 1 shows the estimated precision matrix, fixing the tuning parameters *λ*_1 _= 0.01 and *λ*_2 _= 0.1. It can be seen that $N_{0}^{1}$ is a network with three edges (1, 3), (1, 4), (2, 4), which in the next time point $N_{0}^{2}$ slowly changes to another network with edges (1, 2), (1, 4), (2, 4). In section we consider the full dataset.

**Table 1:** Conditional covariance $\hat{\text{Θ}}$ based on 44 replicates for 4 genes measured across 2 time points. The tuning parameters *λ*_1 _and *λ*_2 _were fixed to 0.01 and 0.1, respectively.

Time		*1*				*2*			
	Gene	*ZNF*	*CCN*	*SIV*	*SCY*	*ZNF*	*CCN*	*SIV*	*SCY*
*1*	ZNF	1.24	0.00	-0.26	0.18	-0.22	-0.11	-0.11	-0.07
	CCN	-	1.49	0.00	-0.17	-0.18	-0.84	0.06	0.12
	SIV	-	-	1.44	0.00	-0.15	0.08	-0.69	-0.01
	SCY	-	-	-	1.19	0.02	0.13	0.41	-0.10

*2*	ZNF	-	-	-	-	1.07	-0.02	0.00	0.12
	CCN	-	-	-	-	-	1.55	0.00	0.24
	SIV	-	-	-	-	-	-	1.52	0.00
	SCY	-	-	-	-	-	-	-	1.08

## Results

### Simulation study: comparison with other methods

In this section we compare the dynamic network inference method with other methods proposed in the literature to estimate networks. [28] suggest a procedure based on large-scale hypothesis testing of partial correlations in combination with false discovery rate cut-offs, implemented in the R-package **GeneNet**. [29] propose an empirical bayes method for estimating biological networks from temporal microarray data. Their method aims to infer a directed graphical model, a so-called Bayesian network, that remains constant through time. This method is implemented in the R-package **ebdbNet**. There is a whole class of methods based on the graphical lasso [20]. Besides the original method, [22] proposed a factorial graphical lasso, implemented in the **sglasso **R-package and [30] consider a sparse autoregressive network inference method using chain graphical models, implemented in the R-package **SparseTSCGM**.

**Figure 1:**
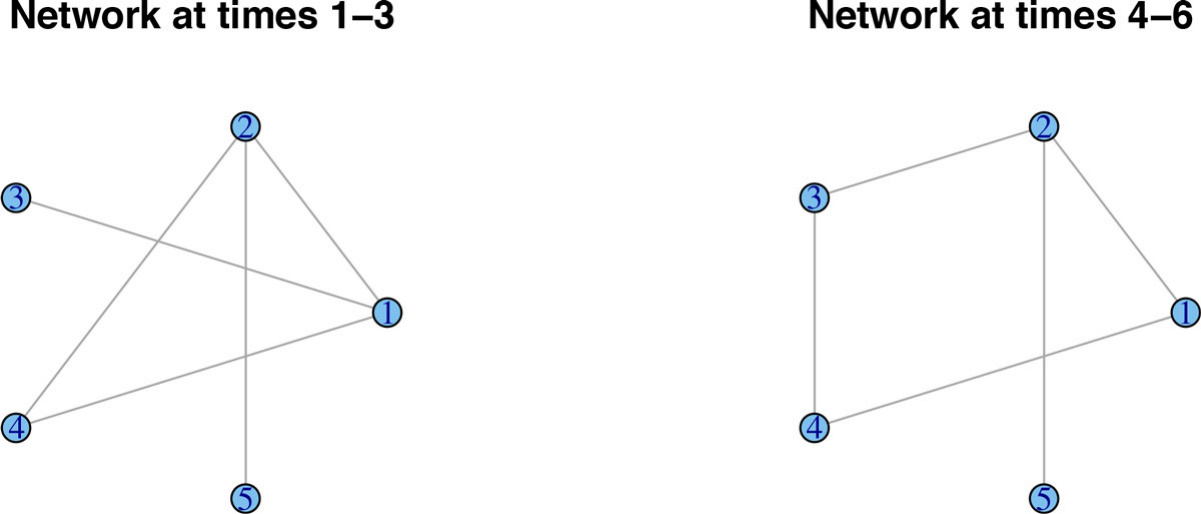
**A regime change between time-points 3 and 4**. Data are simulated from a network that is subject to a regime change between time-points 3 and 4.

**Figure 2:**
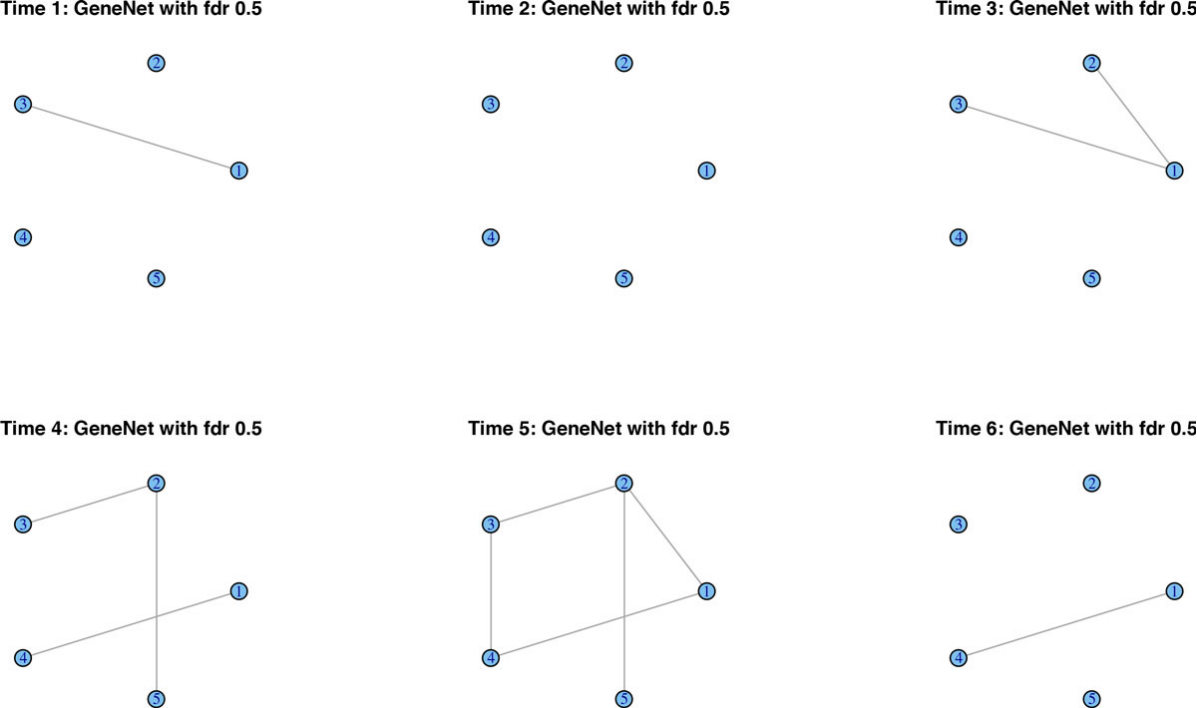
**GeneNet's performance**. GeneNet infers links by means of multiple testing. By lowering the local FDR to 0.5, we recover some of the network structure, but consistency across the time-points is absent.

**Figure 3:**
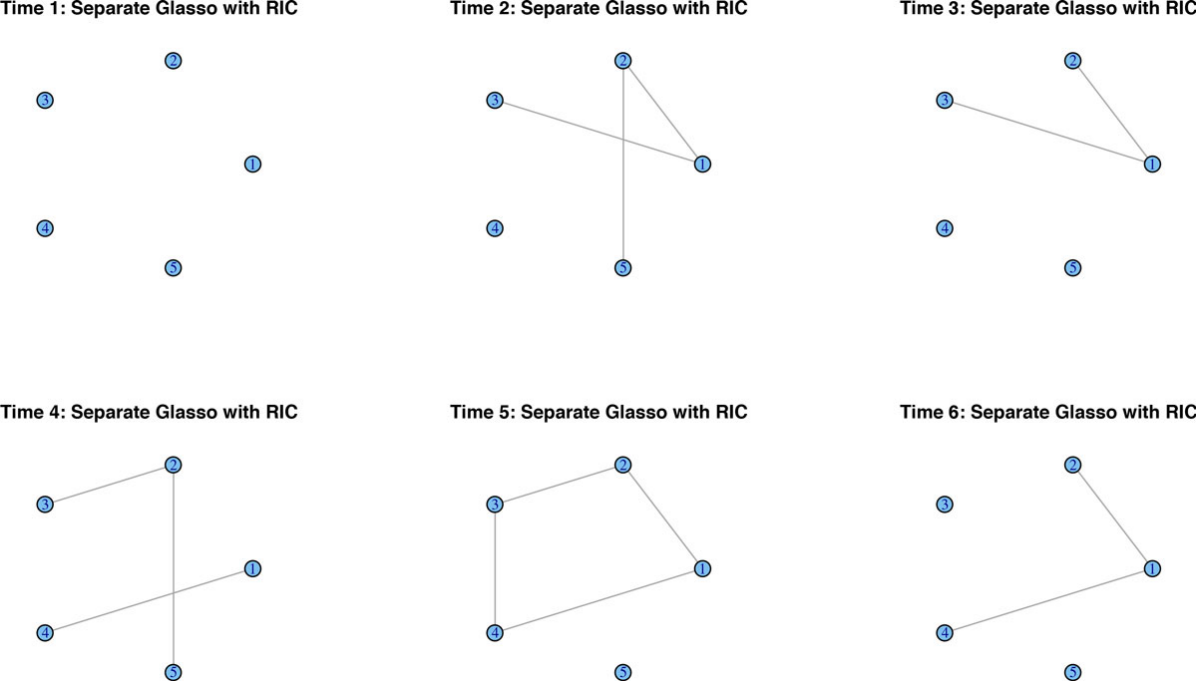
**Graphical lasso's performance**. Graphical lasso estimates a sparse network for each of the time points separately. Although it recovers some of the network structure, there is little consistency across the time-points.

**Figure 4:**
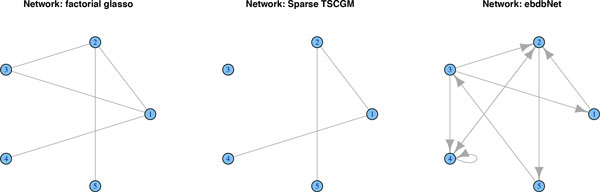
**Performance of Facorial Graphical lasso, Sparse TSCGM and ebdbNet**. Facorial Graphical lasso, Sparse TSCGM and ebdbNet each recover a constant network across the 6 time points. It shows the general underlying structure of the network, but fails to detect the change point.

**Figure 5:**
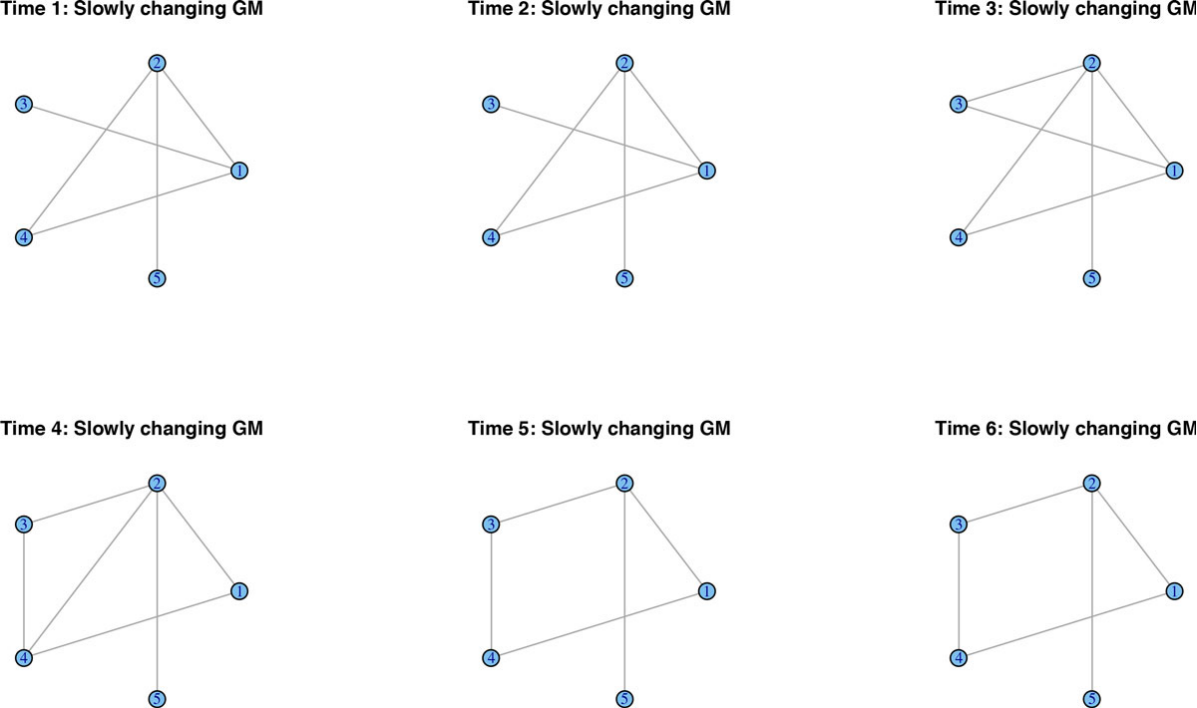
**Performance of the Slowly Changing Graphical model**. The slowly changing graphical model misses some of the timings of the changes, but correctly identifies the regime change and it recovers the underlying structure of the changing network.

We simulate data from a network along six time-points that is affected by a regime change between time points 3 and 4. Figure 1 shows the original networks, interpreted as lag zero conditional independence graphs *N*_0_. We simulate *n *= 100 observations and report the results of the methods described above. Due to the large number of links, GeneNet by default corrects for multiple testing, resulting in a very sparse graph. In fact, it merely detects seven edges throughout the whole time-course, when correcting at the 0.9 local FDR rate. In Figure 2, we lowered the local FDR to 0.5, which allows us to pick up additional edges, but it clearly lacks consistency across the various time points. Roughly the same results crystalize, when applying separate graphical lassos to each of the time-points. The tuning parameter is selected by using the RIC. Figure 3 shows that some structure of the underlying graph has been recovered, but with disappointing consistency across the time-points. Factorial graphical lasso, sparse TSCGM and ebdbNet all infer a constant graph across time, which indeed captures some aspects of the underlying structure, but fails to detect the change point (cf. Figure 4). Although not perfect, the slowly changing graphical model approach correctly borrows strength across the 6 time-points to more accurately infer the underlying graph and at the same time to correctly detect the underlying changes in the dynamics (cf. Figure 5).

### Simulation study: varying network size

We perform a simulation study to show the performance of the autoregressive Gaussian graphical model of order one. We consider four different scenarios with a varying number of genes *p *∈ {20, 40, 60, 80}, each with *n *= 50 observations across *T *= 3 time points. For each scenario we simulate 100 datasets from a multivariate normal distribution with ***μ ***equal to zero and **Σ **equal to the inverse of a precision matrix **Θ**. The structure of the graph slowly changes across time and observations are conditionally independent for time lags greater than one. Note that in all four scenarios the number of replicates *n *is fewer than the number of random variables *pT*.

Table 2 shows the average of false positive, false negative, false discovery, false non-discovery rates as well as the average *F*_1 _score overr 100 simulations. We use the corrected and normal AIC, as well as the BIC to select the tuning parameters in the models. The corrected AIC adds an additional penalty to account for the small number of observations. These results show that the slowly changing autoregressive Gaussian graphical model is very reliable even with small numbers of observations and that it can be used for real applications when few changes in different time points are present using any type of model selection method.

**Table 2:** The average of performance of various model selection algorithms for the four simulation scenarios using four model selection methods in term of the fraction of correctly estimated link/non-links, i.e. false positives (FP), false negatives (FN), false discoveries (FD) and false non-discoveries (FnD), as well as the *F*_1 _= (2 - 2*FN*)/(2 - *FN *+ *FP*) score, which measures the overall average accuracy of recall and precision. The best scores are indicated by bold font.

p		$\bar{FP}$	$\bar{FN}$	$\bar{FD}$	$\bar{FnD}$	*F*_1 _score
20	AICc	**0.0092**	0.0811	**0.2000**	0.0031	0.9532
	BIC	0.0363	0.0139	0.4873	0.0005	**0.9751**
	AIC	0.0698	**0.0069**	0.6470	**0.0003**	0.9628

40	AICc	**0.0057**	0.0447	**0.2899**	0.0006	0.9743
	BIC	0.0088	0.0321	0.3826	0.0005	**0.9793**
	AIC	0.0437	**0.0041**	0.7514	**0.0001**	0.9766

60	AICc	**0.0016**	0.4585	**0.2730**	0.0036	0.7018
	BIC	**0.0016**	0.4585	**0.2730**	0.0036	0.7018
	AIC	0.0288	**0.1452**	0.8088	**0.0012**	**0.9076**

80	AICc	**0.0091**	0.1034	**0.1680**	0.0052	0.9410
	BIC	0.0396	0.0517	0.4527	0.0027	0.9541
	AIC	0.0670	**0.0000**	0.5704	**0.0000**	**0.9675**

### Application to T-cell experiment

**Figure 6:**
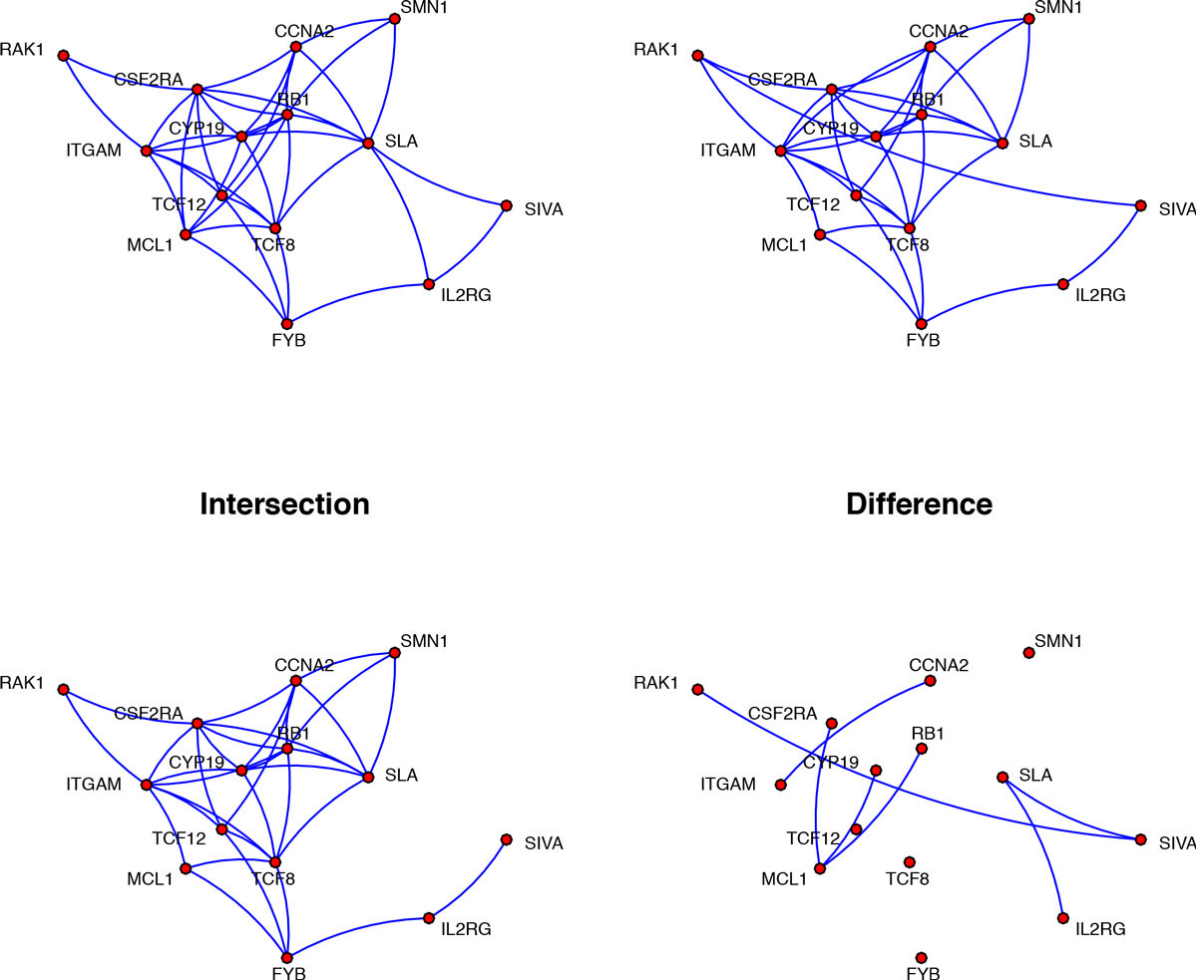
**T-cell network change between *t *= 1 and *t *= 2**. The lag zero network *N*0 for the T-cell data with changes between time points *t *= 1 and *t *= 2.

We apply the autoregressive Gaussian graphical model of order one to the human T-cell dataset. We assume that genes which are two time points apart, i.e. *Y*_*s,t *_and *Y*_*s,t*+2_, are conditional independent given the intervening observations. This means that the edge set for networks at lag 2, i.e. *N*_2_, is an empty set. Figure 6 is obtained from the estimation procedure. The two upper graphs show the two networks at time points 1 and 2, respectively. The bottom left figure, "Intersection," shows the large overlap between the two networks, induced by the significant tuning parameter *ρ*_2_. On the other hand, the bottom right figure shows the changes between these two time points. It shows, for example, that initially MCL1, a pro-survival BCL2 family member, is a highly connected node in the T-cell network. It is known that SCF(FBW7) regulates cellular apoptosis by targeting MCL1 for ubiquitylation and destruction [31]. This is probably why initially MCL1 loses connections to other genes.

## Conclusion

Many time-course genomic experiments are performed in order to discover certain regime changes that may be taking place during that period. Under these circumstances, representing genomic interactions by means of a static graphs can be misleading. Certainly, it would fail to detect any changes in the topology of the network. We propose a sparse dynamic graphical model to infer the underlying slowly changing network. One of the major contributions is that this methodology is capable of providing fast inference about the dynamic network structure in moderately large networks. Until now, even sparse static inference could be painstakingly slow and would typically lack obvious interpretation. We applied the method to a human T-cell dataset to study the developmental aspects of the sparse genomic interactions. One result, backed up by recent research, is that MCL1 is targeted early on and thereby loses its connections to the rest of the genomic network.

Once a graph has been estimated and changes have been evaluated, other questions on how to analyze time-evolution networks might be posed. Does the network retain certain graph properties as it grows and evolves? Does the graph undergo a phase transition, in which its behaviour suddenly changes? In answering these questions it is of interest to have a diagnostic tool for tracking graph properties and noting anomalies and graph characteristics of interest. For example, a useful tool is ADAGE [32], which is a software package that analyzes the number of edges over time, the number of nodes over time, the densification law, the eigenvalues over increasing nodes, the size of the largest connected component, the number of connected components versus nodes and time and the comparative sizes of the connected components over time.

## Competing interests

The authors declare that they have no competing interests.

## Authors' contributions

Both authors have contributed equally to this manuscript.

## Declaration

Publication costs were sourced from the Statistics and Probability research unit inside the Johann Bernoulli Institute at the University of Groningen.

This article has been published as part of *BMC Bioinformatics *Volume 16 Supplement 6, 2015: Selected articles from the 10th International Meeting on Computational Intelligence Methods for Bioinformatics and Biostatistics. The full contents of the supplement are available online at http://www.biomedcentral.com/bmcbioinformatics/supplements/16/S6.
